# Editorial: Inflammatory Treg reprogramming in the tumor immune microenvironment

**DOI:** 10.3389/fimmu.2024.1411923

**Published:** 2024-04-23

**Authors:** Xinpei Deng, Yaorong Su, Hangcheng Fu, Yuzhen Gao, Peng Liu

**Affiliations:** ^1^ State Key Laboratory of Oncology in South China, Guangdong Provincial Clinical Research Center for Cancer, Sun Yat-sen University Cancer Center, Guangzhou, Guangdong, China; ^2^ Jiangmen Central Hospital, Jiangmen, Guangdong, China; ^3^ Department of Urology, University of Louisville School of Medicine, Louisville, KY, United States; ^4^ Department of Clinical Laboratory, Sir Run Run Shaw Hospital of Zhejiang University School of Medicine, Hangzhou, Zhejiang, China

**Keywords:** tumor microenvironment, regulatory T cell (Treg), immunotherapy, inflammatory Treg reprogramming, tumor immunity

Regulatory T (Treg) cells, a subset of immune regulatory cells capable of triggering immunological tolerance, are found within the intricate tumor immune microenvironment (TME) composed of diverse cellular and chemical components (1). Through the suppression of other immune cells, Treg maintain immunological homeostasis and self-tolerance in typical conditions (2). The immunosuppressive activity of Treg serves a dual function in both the initiation and progression of tumors (3, 4).

Recent studies have shown that specific signaling molecules present within the TME can induce reprogramming of inflammatory Treg (5). This reprogramming may lead to a shift of inflammatory Treg from a normal immune regulatory phenotype to one that facilitates tumor progression. Additionally, inflammatory Treg cells are affected by factors produced by various immune cells and tumor cells in the TME. The intratumoural Tregs content exhibits significantly elevated levels compared to those found in peripheral blood or healthy tissue and express checkpoint molecules in high level, rendering them a prime candidate for immunotherapeutic interventions (6). Anti-PD-1/PD-L1 agents are currently employed in immunotherapy to enhance immune-mediated eradication of tumor cells by inhibiting communication between immune and tumor cells (7). These medications can also boost inflammatory Treg activity, which negates their impact on the anti-tumor immune response (8). Consequently, a prominent area of contemporary investigation focuses on the development of innovative tumor immunotherapy strategies to counteract Treg reprogramming. This Research Topic, titled “*Inflammatory Treg reprogramming in the tumor immune microenvironment*”, was curated by 3 guest editors and comprises a collection of 6 articles, including 3 reviews and 3 original research studies. These articles offer a profound comprehension and novel comprehensive perspective on the inflammatory Treg reprogramming in the tumor immune microenvironment and potential strategies for overcoming these challenges.

Treg cells are immunosuppressive CD4+ T cells that are essential for the maintenance of immune tolerance, tissue homeostasis, and prevention of autoimmunity (9). In cancer, it is through these mechanisms that tumors evade the immune system and facilitate progression. The presence of a developing tumor results in tissue damage and inflammatory signals that typically trigger a response from Treg cells. Hence, Treg cells proliferate and amass within the TME as a compensatory mechanism to reestablish immune and tissue equilibrium, ultimately suppressing the immune response against the tumor. Elevated frequencies of intratumoral Treg correlate with poor prognosis in patients with various cancer types, suggesting the potential importance of targeting Treg in cancer immunotherapy (10). In order to facilitate future advancements in the field of Tregs in lung adenocarcinoma (LUAD), Zhang et al. extracted Tregs-associated signatures using single-cell RNA sequencing (scRNA-seq) and bulk RNA-seq. These signatures were subsequently utilized to stratify patient risk subgroups within the context of LUAD. The authors effectively showcased the efficacy of these signatures in assisting the clinical management of LUAD patients. These results have the potential to advance precision medicine and facilitate the identification of new therapeutic targets for LUAD. In another study, Tang et al. conducted a thorough analysis to identify the key factors indicative of the TME and nasopharyngeal carcinoma (NPC), with a specific focus on the role of kinesin family member 18B (KIF18B) in various processes such as TME, epithelial-mesenchymal transition (EMT), N6-methyladenosine (m6A) modification, and therapeutic responses in NPC. The authors successfully demonstrated the effectiveness of KIF18B in predicting patient outcomes, regulating immune evasion and EMT, and predicting therapeutic responses. These findings possess the capacity to propel precision medicine forward and streamline the discovery of novel therapeutic targets for NPC. Moreover, Xu et al. presented a comprehensive analysis of the role of Treg cells infiltration in the immune response of pancreatic cancer. Subsequently, they identified crucial mRNA markers associated with Treg cells in pancreatic cancer. These results offer valuable insights into the potential therapeutic implications of Tregs in cancer treatment.

This Research Topic has also contributed to a profound understanding and novel perspective on inflammatory Treg reprogramming in TME. Treg cells are ubiquitously found within cancer tissues and are crucial for maintaining immune tolerance and tissue equilibrium. The tumor inflammatory microenvironment causes the reprogramming of Tregs, resulting in the conversion of Tregs to immunosuppressive phenotypes. This process ultimately facilitates tumor immune escape or tumor progression. Wu et al. provide a foundation for downregulating the immunosuppressive role of Treg in the inflammatory environment in future tumor immunotherapy. Many recent studies have found that Foxp3+ Treg cells are the major immunosuppressive cells that facilitate the formation of TME by promoting the development of various tumor-associated cells and suppressing the activity of effector immune cells, Riaz et al. demonstrated advances in the immune-suppressive mechanisms of Tregs, the post-translational regulations of Foxp3, and the potential therapeutic targets and strategies to target the Tregs in TME to improve anti-tumor immunity. Meanwhile, Liu et al. elucidated the potential mechanisms involved in Treg cell reprogramming and explores the application of Treg cell immunotherapy. Nevertheless, deeper understandings of the mechanisms that drive Treg cell reprogramming are warranted to ascertain its suitability for future clinical applications.

In summary, this Research Topic encompasses a comprehensive examination and analysis of 6 articles that delve into the topics of inflammatory Treg reprogramming in the tumor immune microenvironment. These articles present diverse perspectives on the subject matter, providing valuable insights and potential strategies to improve the effectiveness of immunotherapy. Treg, as a prominent target in the realm of cancer immunotherapy, has the potential to collaboratively stimulate a strong anti-tumor response, particularly in instances where tumors have shown resistance to immunotherapy. By modulating the reprogramming of Treg cells, it is feasible to diminish their immunosuppressive activity, alleviate the adverse impacts of Treg reprogramming on immunotherapy in inflammatory settings, and partially avert the severe autoimmune repercussions that could result from Treg depletion therapy. We believe that in the future, with continued in-depth research on Treg, s substantial advancements will be achieved in the realm of tumor immunotherapy, ultimately benefiting a large population of individuals with cancer. It is our aspiration that the findings outlined in this Research Topic will serve as a driving force for future innovations, fostering an stimulating and auspicious future in this field.

## Author contributions

XD: Writing – original draft. YS: Writing – original draft. HF: Writing – review & editing. YG: Writing – review & editing. PL: Writing – review & editing.

## References

[1] Tay C Tanaka A Sakaguchi S. Tumor-infiltrating regulatory T cells as targets of cancer immunotherapy. Cancer Cell. (2023) 41:450–65. doi: 10.1016/j.ccell.2023.02.014 36917950

[2] Ohkura N Kitagawa Y Sakaguchi S. Development and maintenance of regulatory T cells. Immunity. (2013) 38:414–23. doi: 10.1016/j.immuni.2013.03.002 23521883

[3] Tanaka A Sakaguchi S. Regulatory T cells in cancer immunotherapy. Cell Res. (2017) 27:109–18. doi: 10.1038/cr.2016.151 PMC5223231 27995907

[4] Li Y Zhang C Jiang A Lin A Liu Z Cheng X. Potential anti-tumor effects of regulatory T cells in the tumor microenvironment: a review. J Transl Med. (2024) 22:293. doi: 10.1186/s12967-024-05104-y 38509593 PMC10953261

[5] Lian X Yang K Li R Li M Zuo J Zheng B. Immunometabolic rewiring in tumorigenesis and anti-tumor immunotherapy. Mol Cancer. (2022) 21:27. doi: 10.1186/s12943-021-01486-5 35062950 PMC8780708

[6] Zhulai G Oleinik E. Targeting regulatory T cells in anti-PD-1/PD-L1 cancer immunotherapy. Scand J Immunol. (2022) 95:e13129. doi: 10.1111/sji.13129 34936125

[7] Pang K Shi ZD Wei LY Dong Y Ma YY Wang W. Research progress of therapeutic effects and drug resistance of immunotherapy based on PD-1/PD-L1 blockade. Drug Resist Updat. (2023) 66:100907. doi: 10.1016/j.drup.2022.100907 36527888

[8] Ho P Cahir-McFarland E Fontenot JD Lodie T Nada A Tang Q. Harnessing regulatory T cells to establish immune tolerance. Sci Transl Med. (2024) 16:eadm8859. doi: 10.1126/scitranslmed.adm8859 38478632

[9] Kang JH Zappasodi R. Modulating Treg stability to improve cancer immunotherapy. Trends Cancer. (2023) 9:911–27. doi: 10.1016/j.trecan.2023.07.015 PMC13390109 37598003

[10] Nishikawa H Sakaguchi S. Regulatory T cells in tumor immunity. Int J Cancer. (2010) 127:759–67. doi: 10.1002/ijc.25429 20518016

